# Novel compound heterozygous variants in the *PCCB* gene causing adult-onset propionic acidemia presenting with neuropsychiatric symptoms: a case report and literature review

**DOI:** 10.1186/s12920-022-01202-2

**Published:** 2022-03-16

**Authors:** Yingxuan Li, Miaomiao Wang, Zhaoyang Huang, Jing Ye, Yuping Wang

**Affiliations:** Department of Neurology, Xuanwu Hospital, Capital Medical University, The Beijing Key Laboratory of Neuromodulation, No.45 Changchun Street, Beijing, 100053 China

**Keywords:** Adult-onset propionic acidemia, Neuropsychiatric symptoms, *PCCB* gene, Compound heterozygous mutation, Case report

## Abstract

**Background:**

Propionic acidemia (PA) is a rare autosomal recessive disorder of metabolism caused by mutations in the *PCCA* or *PCCB* gene, leading to propionyl CoA carboxylase (PCC) enzyme deficiencies. Most PA patients present variable clinical phenotypes and severity in the neonatal or infant period, with only a few developing symptoms after infancy. This report describes a PA patient with an adult-onset phenotype and a novel compound heterozygous mutation in the *PCCB* gene. To further explore the genotype–phenotype correlations in late-onset PA, we performed a literature review focusing on and summarizing 11 patients with PCC gene mutations who had the first onset and/or the definite diagnosis after infancy.

**Case presentation:**

A 21-year-old PA patient presented with weakness of four limbs, gait abnormalities, two episodes of seizures, mental and behavior disorders after severe vomiting. Magnetic Resonance Imaging (MRI) demonstrated sustained bilateral caudate head and putamen symmetrical hyperintensity. Biochemical investigations revealed plasma amino and urine values correlating with a PA profile. Genetic analysis confirmed novel compound heterozygous variants in *PCCB*, with a newly-found pathogenic mutation (c.467T>C) and the c.1316A>G mutation associated with pathogenicity.

**Conclusion:**

We identified a novel compound heterozygous mutation in the *PCCB* gene causing late-onset PA. Patients carrying mutations in the *PCCB* gene tend to develop late-onset PA and present neuropsychiatric symptoms and/or signs. Further molecular biological research is needed to explore the genotype–phenotype correlations of PA.

**Supplementary Information:**

The online version contains supplementary material available at 10.1186/s12920-022-01202-2.

## Background

Propionic acidemia (PA) (OMIM#606054) is a rare autosomal recessive disorder of metabolism caused by mutations in the *PCCA* or *PCCB* gene, which leads to deficiencies of the propionyl CoA carboxylase (PCC) enzymes [1]. The global incidence of PA is estimated to be 1:50,000 to 1:100,000 [2]. PA can be classified as either neonatal (younger than 3 months) or late-onset (older than 3 months), depending on the age of onset [3]. The neonatal form is typically presented with vomiting, refusal to feed, hypotonia, seizure, coma, and other symptoms. In contrast, the clinical manifestations of late-onset PA are non-specific, including intellectual disability, optic atrophy, dilated cardiomyopathy, pancreatitis, renal failure, and premature ovarian failure. Furthermore, patients with late-onset PA tend to have better long-term survival, while neonatal patients have a worse prognosis [4, 5].

This case report describes an adult female patient with novel compound heterozygous variants in the *PCCB* gene presenting neuropsychiatric symptoms and bilateral basal ganglia hyperintensity with magnetic resonance imaging (MRI). With the confirmation of carrying a previously reported pathogenic mutation (c.1316A>G) and a novel pathogenic mutation (c.467T>C) in the *PCCB* gene, the patient was finally diagnosed with late-onset PA.

## Case presentation

**Fig. 1:**
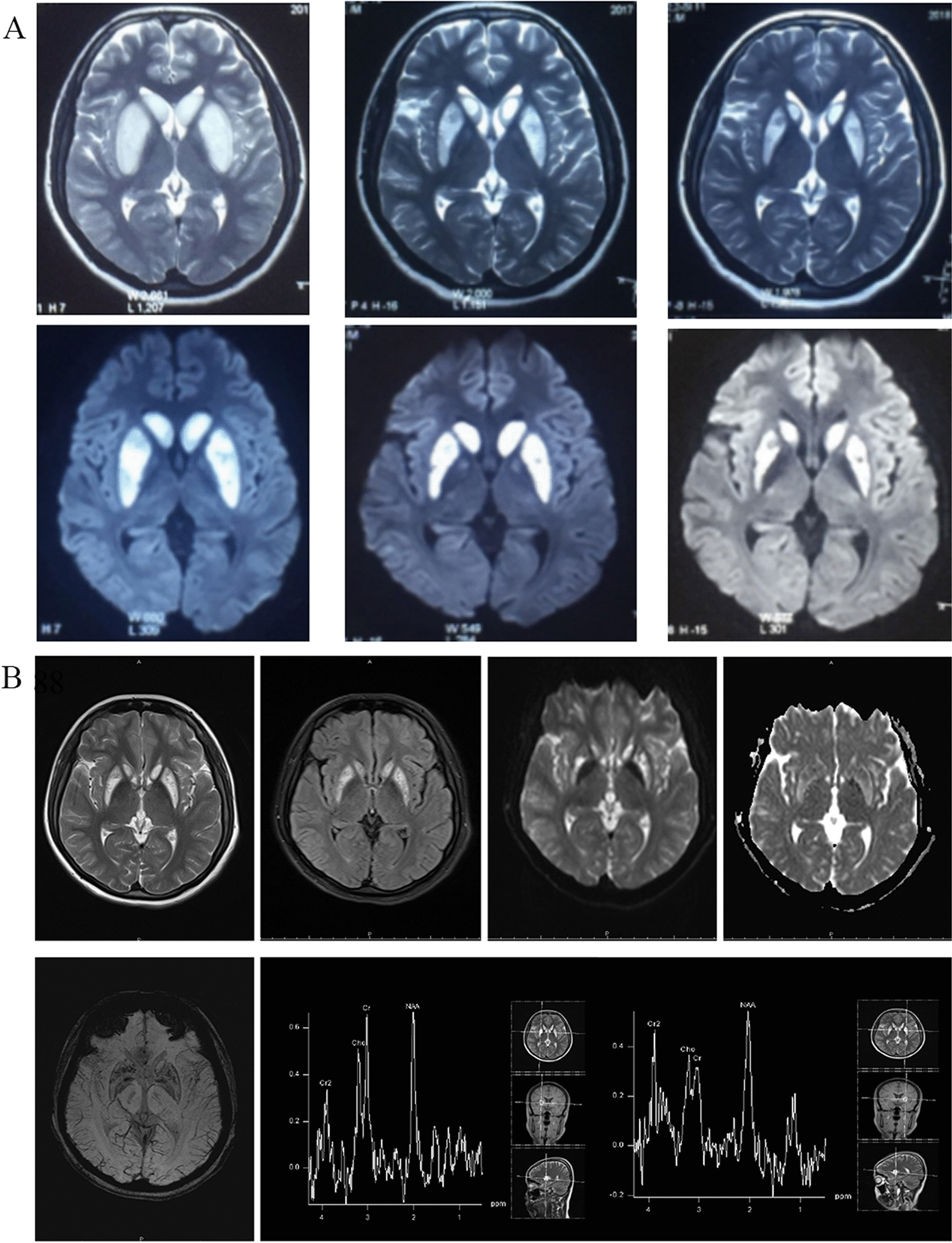
The comparison of imaging findings at different times. **A** Images of T2 weighted imaging (T2WI) (the top row) and diffusion-weighted imaging (DWI) (the bottom row) in the local hospital at different times (2 days, 27 days, and 3 months after onset, respectively) showed symmetrical hyperintensity of bilateral basal ganglia nuclei. **B** Comparison of MRI imaging at different sequences in our hospital, including T2WI, T2-fluid attenuated inversion recovery (FLAIR), DWI, apparent diffusion coefficient (ADC), susceptibility-weighted imaging (SWI) sequences, and magnetic resonance spectrum (MRS), respectively. The radiologic findings showed hyperintensity of the bilateral caudate head and putamen in T2WI, FLAIR, and DWI, which were associated with scattered hypointensity in ADC and SWI. MRS showed decreased NAA in bilateral putamens with the emergence of a lipid peak in the left side

The patient was a 21-year-old Chinese woman who was the first of three siblings in a non-consanguineous marriage, with a normal perinatal period and negative family history of PA. She started school at an appropriate age and graduated from secondary school. She had an unplanned pregnancy at the age of twenty and suffered from severe vomiting at thirteen weeks of gestation. She then developed weakness and was unable to walk steadily. After suffering from frequent vomiting for another two days, she decided to perform a drug abortion. She suddenly began shouting loudly the following day and could not communicate coherently with others. Subsequently, she was admitted to the local hospital after two consecutive seizures, presenting as tics of four limbs and losing consciousness. Each seizure episode lasted about one minute, and the total interval was five minutes. No seizure occurred after treatment with phenobarbital, but she developed a state of mutism after that. Laboratory findings revealed deficiencies of vitamin B12 and vitamin D. T2 weighted imaging (T2WI) showed bilateral hyperintensity of basal ganglia, associated with mixed-signal intensities using diffusion-weighted imaging (DWI) sequences (Fig. 1A). Following these analyses, the patient was tentatively diagnosed with Wernicke encephalopathy. Therefore, she received oral antiepileptic drugs and folate tablets for one week, combined with vitamin B1 and vitamin B12 injections. Her mental symptoms improved and her brain MRI revealed smaller lesions. However, she still was unable to walk steadily.

Four months following discharge, she began presenting frequent and inappropriate uncontrollable laughing without convulsions or loss of consciousness, which would last for one minute. One month later, she was admitted to our hospital in May 2018. Neurological examination showed short-term memory loss, count disturbance, uncontrollable left-beating horizontal nystagmus, gait abnormalities, and decreased sensation below bilateral wrist joints. Muscle strength and tendon reflex were normal and Babinski sign was negative. Brain MRI still showed bilateral caudate head and putamen hyperintensity in T2WI, and T2-fluid attenuated inversion recovery (FLAIR) imaging, associated with mixed-signal DWI and weak scattered signals in apparent diffusion coefficient (ADC) and susceptibility-weighted imaging (SWI) sequences. Magnetic resonance spectrum (MRS) showed decreased N-acetylaspartate (NAA) in bilateral putamens with the emergence of a lipid peak on the left side (Fig. 1B). Her electrocardiogram demonstrated a sinus rhythm with 68 beats/min and a prolonged QT interval (QTc = 476 ms).

In addition, her visual acuity was 20/400 in the right eye and 20/63 in the left eye. Bilateral optic atrophy was identified by optical coherence tomography. Laboratory testing revealed a continued deficiency of vitamin B12. Urine organic acid tests using gas chromatography-mass spectrometry (GS-MS) revealed increases in 3-hydroxypropionate and the presence of methylcitrate, 3-methyl-paracylglycine, paracylglycine, and propionylglycine. Furthermore, propionylcarnitine and C3/C2, C3/C0 levels were also elevated in the plasma amino acid profile.

**Fig. 2:**
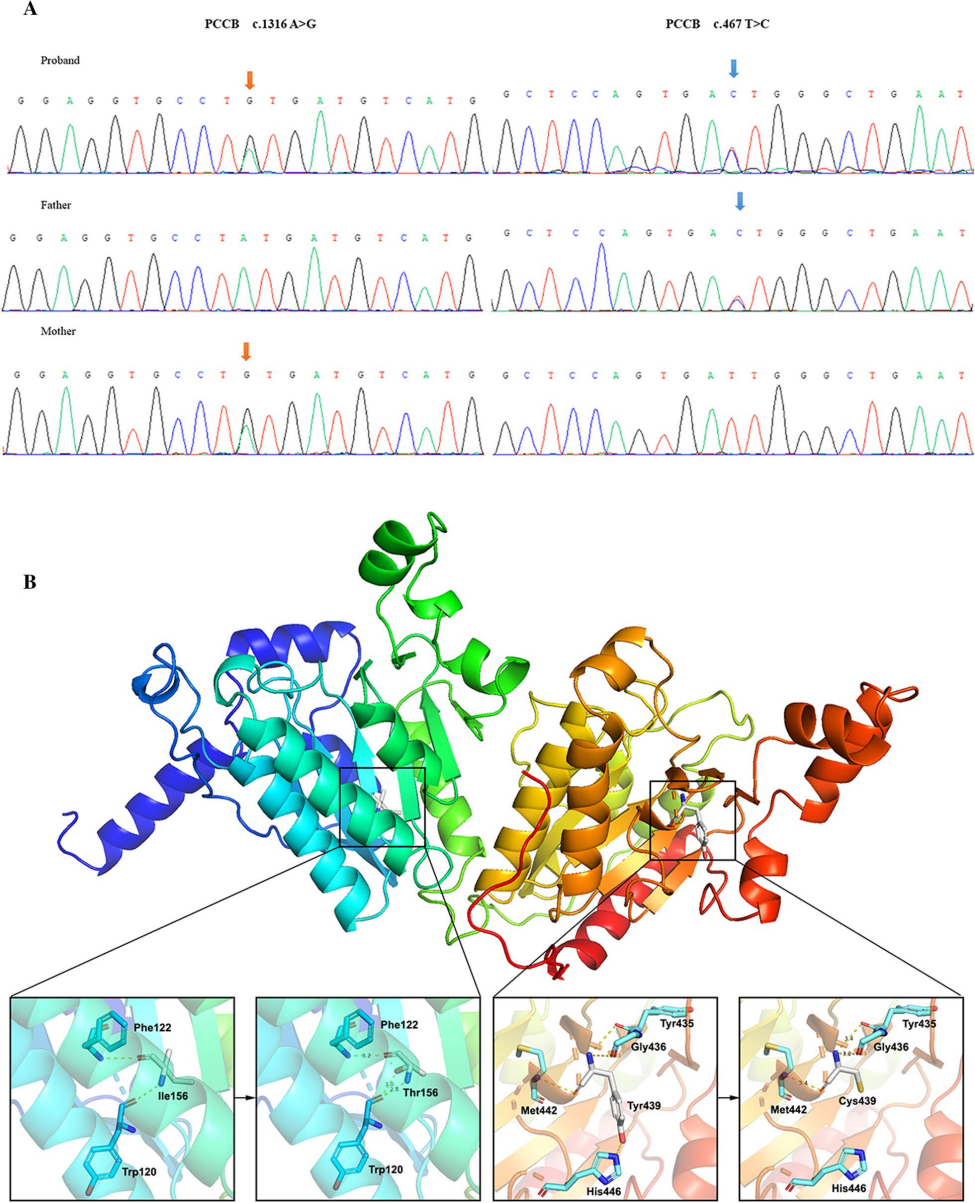
Gene sequencing results and three-dimensional structure model of the PCCB protein. **A** Gene sequencing showed the c.1316A>G mutation (solid orange arrow) in the proband and her mother and the c.467T>C mutation (solid blue arrow) in the proband and her father. **B** Three-dimensional structure modeling of the PCCB protein with isoleucine 156 and tyrosine 439 framed. Polar interactions between the mutation point and surrounding amino acids are shown. Their interactions were maintained before and after the I156T alteration. However, the variant may affect the stabilization of the beta-sheet. The interaction between His446 and Tyr439 is disrupted when tyrosine is altered to cysteine, leading to the structural instability of the whole protein

Whole-exome sequencing for the patient was conducted with a 120.620X average sequencing depth, and 99.80% mean coverage. Genetic analysis revealed compound heterozygous mutations in the *PCCB* gene: c.1316 A>G (p.Tyr 439 Cys) and c.467T>C ( p.Ile 156 Thr), which were inherited from the patient’s mother and father, respectively (Fig. 2A). Notably, the c.1316A>G mutation has been previously reported to be pathogenic. In contrast, the c.467T>C mutation is the first to be reported here, which is absent from controls (1000 Genomes, ExAC, gnomAD, and CNGB) and is *in trans* with the pathogenic mutation c.1316A>G (p.Tyr439Cys). Multiple computational software programs predict that the c.467T>C mutation is likely deleterious. Mutation Taster, Provean, SIFT, and Polyphen-2 predicted the c.467T>C mutation as disease-causing (probability 0.999), deleterious (score − 4.77 <  − 2.5), damaging (score 0.000 < 0.05) and/or probably damaging (score = 1.000), respectively (Supplementary data). The entire structure of the PCC enzyme was demonstrated according to the protein sequence (NP_000523) by SWISS-MODEL (https://swissmodel.expasy.org/). The polar interactions between the mutation points and surrounding amino acids changed drastically with the specified mutations (Fig. 2B). Additionally, the residue p.I156T site was highly conserved across different species. Therefore, the novel c.467T>C mutation is classified as a likely pathogenic mutation according to the American College of Medical Genetics and Genomics (ACMG) standard. The process of whole-exome sequencing and assessing the pathogenicity of the novel mutation for the patient is included in the supplementary data [see Additional file 1 and Additional file 2, respectively].

According to the guideline[4], treatment principles of PA include dietary management (low-protein diet, gastrostomy tube placement with parenteral nutrition), medications (carglumic acid, biotin, antibiotics), and liver transplantation. The patient’s neuropsychiatric symptoms improved gradually after modifications of protein restriction and oral supplementation of folate, biotin, and carnitine.

## Discussion and conclusions

In this case report, we discuss a case of a 21-year-old woman who suffered acute neuropsychiatric symptoms after severe vomiting. Her brain MRI was characterized by bilateral caudate head and putamen hyperintensity on T2WI and DWI sequences. Genetic analysis showed that she harbored compound heterozygous mutations in the *PCCB* gene, with a newly-found pathogenetic mutation (c.467T>C) and a mutation (c.1316 A>G) with known pathogenicity.

Our patient showed acute deterioration of cognition and mental state combined with two seizure episodes. As previously described, seizure, dystonic movements, cognitive impairment, and developmental delay are common neurological manifestations of late-onset PA. Psychiatric symptoms such as irritability, panic, hallucinations, massive anxiety, and grossly disorganized behaviors have also been described [6, 7]. Complex mechanisms, including neurotransmitter pathway disturbances and accumulation of toxic products, can result in epileptogenesis and neuronal malfunction. Therefore, metabolic treatments combined with antiepileptic drugs may prevent the recurrence of seizures [6].

Typical brain MRI signal abnormalities of the basal ganglia in late-onset PA include hyperintensity of bilateral putamen, globus pallidus, and the caudate head in T2WI, FLAIR, and DWI sequences [6, 7]. Our patient presented similar image findings. However, these findings lasted more than six months. In our case, a decreased NAA peak in bilateral putamens indicated neuronal damage, and the lipid peak in the left putamen demonstrated necrosis in the lesion.

In this case report, a novel compound heterozygous mutation was identified. The c.1316 A>G mutation in the *PCCB* gene has been reported earlier and is pathogenic [8–10]. The novel pathogenetic mutation c.467T>C identified in this report was not found in the Human Gene Mutation Database (http://www.hgmd.org). Likewise, there is no functional evidence for this variation in ClinVar.

(https://www.ncbi.nlm.nih.gov/clinvar/variation/VCV000570186.1). Typically, most patients with PA present symptoms in the neonatal and infant period. Only a few PA patients develop symptoms after infancy, perhaps even asymptomatic until adulthood [4, 5]. Due to this variability in presentation, it is challenging to diagnose late-onset PA, especially in adult patients. Therefore, to further explore the genotype–phenotype correlations in PA patients with the first onset and/or definite diagnosis after infancy (12 months of age), we performed a literature review and summarized another 11 cases of late-onset PA patients with onset after infancy and identified associated PCC gene mutations (Table 1). The age of onset and/or diagnosis was between 14 months to 27 years old. We found that the most common initial clinical manifestation among these patients was neuropsychiatric disorders (8/11, 72.7%). Thirteen different *PCCB* gene mutations and one *PCCA* gene mutation were identified, of which some patients had the same mutant allele (PAT.3 and PAT.4 had c.1606 A>G in *PCCB*, PAT.5 and PAT.6 had c.1316 A>G in *PCCB*, PAT.8 and PAT.10 had c.1229 G>A in *PCCB*). Our findings demonstrate the predominance of *PCCB* gene mutations (10/11, 90.9%) in late-onset PA patients presenting symptoms after infancy, consistent with findings from McCrory’s study [19].

**Table 1:** Summary of 12 PA patients with the first onset/diagnosis after infancy

PAT	Ref.	Age of onset/diagnosis	Sex	Symptoms/signs related to PA			Image findings		Electrophysiologic findings			Genotype			Treatments	Follow-up
				Neurological	Gastrointestinal	Circulatory	UCG	Brain-MRI	ECG	EEG	Gene	Mutation site	Homo	Hetero		
1	This case	20y; 20y	F	ataxia; cognitive impairment; seizure	N	N	NA	Hyperintensity of bilateral head of caudate and putamen	Sinus rhythm; prolonged QTc interval	NA	PCCB	c.467T>C; c.1316A>G	N	Y*	Protein restriction; vitamin B1, B12, folic acid, biotin and carnitine	Symptoms improve
2	[11]	1.5y; 10.5y	F	N	Feding refusal; vomiting	N	NA	NA	NA	NA	PCCA	c.424C>A	Y	N	NA	NA
3	[12]	3y; 3y	F	Encephalopathy	Abdominal pain; vomiting	N	Normal biventricular size and function	NA	Sinus rhythm; prolonged QTc iinterval	NA	PCCB	c.1606A>G	Y	N	Protein restriction and intravenous nutrition; L-carnitine	No neurologic deficits
4	[12]	27y; 27y	F	NA	N	DCM; cardiogenic shock	Left ventricular hypertrophy: LVEF was 15%	NA	NA	NA	PCCB	c.1606A>G	Y	N	Dietary management; L-carnitine; ECMO; heart transplantation	Followed in the biochemical genetics outpatient clinic
5	[8]	3y; 4y	M	Fully conscious; generalized tonic convulsion	Acute pancreatitis	Decreased blood pressure	Decreased left ventricular contractility, LVEF was 41.0%	NA	Tachycardia; prolonged QTc interval	NA	PCCB	c.1316A>G; exon 8 deletion	N	Y*	TPN fluid without amino acid; MPA formula; metronidazole; antioxidant cocktail	Normal growth and development
6	[9]	2y; 2y	M	Developmental delay; learning disabilities	N	Cardiac arrest	Reduced LVEF	NA	Normal	NA	PCCB	c.1316A>G; c.331C>T	N	Y*	Low-protein diet, smultivitamin, L-carnitine; resuscitation and support	Symptoms improve
7	[13]	14 m; 14 m	M	Developmental delay	N	N	NA	NA	NA	NA	PCCB	c.1210G>A	Y	N	NA	Normal development
8	[14]	16y; 16y	M	N	N	Tachycardic; systolic murmur;; dyspnoea; exercise intolerance;	DCM	NA	Prolonged QT cinterval	NA	PCCB	c.1229G>A	Y	N	Diuretics; inotropes	Rapid recovery within 2 months
9	[15]	1y; 8y	M	Lethargy	N	N	NA	NA	NA	NA	PCCB	c.49C>A	Y	N	Protein restriction; carnitine,biotin, thiamine;	Passed away 1 week after tracheostomy
10	[16]	14y; 14y	M	Exercise intolerance	Nausea and diarrhea, hepatomegaly, splenomegaly	Bigeminy, S3 gallop; poor peripheral pulses; prolonged capillary refill time;	Dilated left ventricle with diminished function; poor right ventricular function	NA	NA	NA	PCCB	c.763 + 2 T>G; c.1229G>A	N	Y*	Cardiac transplantation	Rapid recovery after cardiac transplantation
11	[17]	4.5y; 4.5y	F	Comatose	N	N	NA	NA	NA	Generalized delta activity	PCCB	L417W; Q293E	N	Y*	Protein restriction, high-caloric nutrition; biotin carnitine, correction of acidosis	NA
12	[18]	5y; 5y	M	Dystonic movements; coma; hypotonia; dysarthria;	Vomiting	N	NA	Bilateral symmetrical hyperintensity and enlargement of basal ganglia	NA	Nonspecic slowing	PCCB	IVS10-11del6; c.1228C>T	N	Y*	Protein restriction; carnitine, biotin;	Died of cardiac arrest

M: Male; F: Female; Y: Yes; N: No; NA: not available; d: day; m: month; y: years; Ref.: reference; CSF: cerebrospinal fluid; DIC:disseminated intravascular coagulation; DCM: dilated cardiomyopathy; UCG:ultrasonic cardiogram; LVEF: left ventricular ejection fraction; ECMO: extracorporeal membrane oxygenation; ICU: intensive care unit; TPN: total parenteral nutrition; MPA: methylmalonic propionic acidemia; Homo: Homozygous; Hetero: Heterozygous

*Compound heterozygous mutation

According to a classification based on the residual activity of PCC with pathogenic mutations, most *PCCA* variants are classified as destabilizing mutations resulting in a null or deficient residual activity. In contrast, *PCCB* variants tend to have more variable functional outcomes presenting a broader range of phenotypes [20]. These findings may partly explain why patients with *PCCA* mutations may have earlier onset and more severe phenotypes than patients with *PCCB* variants.

In conclusion, we describe a PA patient with a late-onset phenotype and a novel compound heterozygous mutation in the *PCCB* gene. Although we postulated that PA patients carrying mutations in the *PCCB* gene tend to develop late-onset phenotype presenting neuropsychiatric symptoms, further molecular research is needed to explore the genotype–phenotype correlations and validate the pathogenicity of this newly identified mutation.

## Supplementary Information

**Additional file 1.** The process of whole-exome sequencing for the patient.

**Additional file 2.** The process of assessing the pathogenicity of the novel mutation for the patient.

## Data Availability

The clinical case records of our patients are available from the corresponding author on reasonable request. The raw sequencing data of whole-exome sequencing for the patient is available in the National Center for Biotechnology Information (NCBI) Sequence Read Archive (SRA) repository, accession number PRJNA802806 (https://www.ncbi.nlm.nih.gov/sra/PRJNA802806).

## Abbreviations

PA: Propionic acidemia
PCC: Propionyl CoA carboxylase
T2WI: T2 weighted imaging
DWI: Diffusion-weighted imaging
FLAIR: T2-fluid attenuated inversion recovery
ADC: Apparent diffusion coefficient
SWI: Susceptibility-weighted imaging
MRS: Magnetic resonance spectrum
NAA: N-acetylaspartate
GS-MS: Gas chromatography-mass spectrometry
