# Supplementary material for: Novel compound heterozygous variants in the PCCB gene causing adult-onset propionic acidemia presenting with neuropsychiatric symptoms: a case report and literature review
Source: BMC Med Genomics. 2022 Mar 16;15:59. doi: 10.1186/s12920-022-01202-2 (PMC8925091; doi:10.1186/s12920-022-01202-2)
Supplement: Supplementary file 1 — Additional file 1. The process of whole-exome sequencing for the patient. [file 12920_2022_1202_MOESM1_ESM.docx]

**Supplementary data for**

**Novel Compound Heterozygous Variants in *PCCB* Gene Causing Adult-onset Propionic Acidemia Presenting with Neuropsychiatric Symptoms: A Case Report and Literature Review**

Yingxuan Li, Miaomiao Wang, Zhaoyang Huang^*,#^, Jing Ye^*,#^ ,Yuping Wang

*: Corresponding author: Zhaoyang Huang, MD. Jing Ye, MD.

#: Authors Z. Huang and J. Ye contributed equally to this work.

This file contains the process of Whole-exome sequencing for our patient.

**SAMPLES**

DNA was isolated from peripheral blood using DNA Isolation Kit (Blood DNA Kit V2 ，CW2553). Concentrations were determined on a Qubit fluorometer (Invitrogen, Q33216) using Qubit dsDNA HS Assay Kit (Invitrogen, Q32851). Agarose gel (1%) electrophoresis was performed for quality control.

1 μg of the isolated DNA was sheared using the following the parameters: duty cycle 10%, intensity 5, cycles per burst 200, time 6 cycles per 60 seconds. This step was performed on a Bioruptor UCD-200 (Diagenode). For all samples, shearing worked very consistently and the size distribution peak was around 200 bp. 3 μL of the sheared DNA was electrophoresed in a 2% agarose gel to confirm the presence of fragments of the desired size range.

**Library Preparation**

DNA libraries were prepared with KAPA Library Preparation Kit (Kapa Biosystems, KR0453) following the manufacturer’s instructions. The procedure comprises three standard steps: end-repair of fragmented DNA, A-tailing, adapter ligation and amplification. Purifications between steps were carried out with Agencourt AMPure XP beads. The libraries were estimated with Qubit dsDNA HS Assay kit (Invitrogen, Q32851).

1. End Repair Reaction

Assemble each end repair reaction as follows:

| 10× KAPA End Repair Buffer | 4.5 µl |
| --- | --- |
| KAPA End Repair Enzyme Mix | 2.5 µl |
| Fragmented, double-stranded DNA | 35 µl |
| Water | 3 µl |
| Total reaction volume | 45 µl |

Mix, and incubate at 20 °C for 30 min.

Proceed immediately to End Repair Cleanup, as follows:

| End repair reaction | 45 µl |
| --- | --- |
| Agencourt® AMPure® XP reagent | 80 µl |
| Total volume per tube | 125 µl |

Mix thoroughly by pipetting up and down multiple times, and incubate the tube at 37℃ for 2 min. Place the tube on a magnet to capture the beads, incubate until the liquid is clear, and carefully remove and discard the supernatant. Keeping the tube on the magnet, add 200 µl of 80% ethanol, incubate the tube at room temperature for 30 sec, carefully remove and discard the ethanol, repeat this step. Allow the beads to dry at room temperature, sufficiently for all the ethanol to evaporate.

1. A-Tailing Reaction

Add 50µl of A-Tailing Master Mix (10× KAPA A-Tailing Buffer 5 µl, KAPA A-Tailing Enzyme 3 µl, Water 42 µl) to the tube containing beads with end-repaired DNA, and incubate at 30 °C for 30 min. Proceed immediately to A-Tailing Cleanup.

| 10× KAPA A-Tailing Buffer | 2.5 µl |
| --- | --- |
| KAPA A-Tailing Enzyme | 1.5 µl |
| Water | 15 µl |
| Total volume per tube | 25 µl |

Add 45 µl of PEG/NaCl SPRI® Solution to the tube containing the 25 µl A-tailing reaction with beads, mix and incubate the tube at 37℃ for 2 min. Place the tube on a magnet to capture the beads, incubate until the liquid is clear, and carefully remove and discard the supernatant. Keeping the tube on the magnet, add 200 µl of 80% ethanol, incubate the tube at room temperature for 30 sec, carefully remove and discard the ethanol, repeat this step. Allow the beads to dry at room temperature, sufficiently for all the ethanol to evaporate.

1. Adapter Ligation Reaction

Add 45 µl of Ligation Master Mix and 5 µl of Adapter to the tube containing the dried beads with A-tailed DNA, mix, and incubate at 20 °C for 15 min. Proceed immediately to Adapter Ligation Cleanup.

| 5× Kappa Ligation buffer | 5 µl |
| --- | --- |
| Kappa T4 DNA ligase | 2.5 µl |
| Adapter | 2.5 µl |
| Water | 15 µl |
| Total volume per tube | 25 µl |

Add 50 µl of PEG/NaCl SPRI® Solution to the tube containing the 50 µl ligation reaction with beads, mix and incubate the tube at 37℃ for 2 min. Place the tube on a magnet to capture the beads, incubate until the liquid is clear, and carefully remove and discard the supernatant. Keeping the tube on the magnet, add 200 µl of 80% ethanol, incubate the tube at room temperature for 30 sec, carefully remove and discard the ethanol, repeat this step. Allow the beads to dry at room temperature, sufficiently for all the ethanol to evaporate.

1. Library Amplification Reaction

Thoroughly resuspend the beads in 25 µl of water, incubate the tube at room temperature for 2 min. Place the tube on a magnet to capture the beads, incubate until the liquid is clear, transfer the clear supernatant to a new tube and proceed with library amplification.

Assemble each library amplification reaction as follows:

| Library DNA | 10 µl |
| --- | --- |
| 2× KAPA HiFi HotStart ReadyMix | 12.5 µl |
| PCR Primer Premix (5 µM each primer) | 1 µl |
| Water | 1.5 µl |
| Total volume per tube | 25 µl |


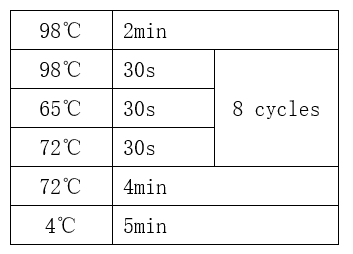
Perform PCR with the thermocycling parameters:

Add 45 µl of Agencourt^®^ AMPure^®^ XP reagent to the tube containing the 25 µl ligation reaction with beads, mix and incubate the tube at 37℃ for 2 min. Place the tube on a magnet to capture the beads, incubate until the liquid is clear, and carefully remove and discard the supernatant. Keeping the tube on the magnet, add 200 µl of 80% ethanol, incubate the tube at room temperature for 30 sec, carefully remove and discard the ethanol, repeat this step. Allow the beads to dry at room temperature, sufficiently for all the ethanol to evaporate. Thoroughly resuspend the beads in 25 µl of water, incubate the tube at room temperature for 2 min. Place the tube on a magnet to capture the beads, incubate until the liquid is clear, transfer the clear supernatant to a new tube, and proceed with array capture.

**Array capture**

Hybridization of pooled libraries to the capture probes and removal of non-hybridized library molecules were carried out according to the SeqCap hybrid Mix system

The capture array hybridization method involves mixing the pooled product with a buffer solution, then incubate the hybridization mixture for 20 hours at 65°C.

| SeqCap 2×Hybridization Buffer | 8.5 µl |
| --- | --- |
| SeqCap Hybridization Component A | 3.4 µl |
| Nuclease-Free Water | 1.1 μL |
| probe | 4 μL |
| Nuclease-Free Water | 3 μL |
| Total volume per tube | 20 µl |

Library molecules fished out by hybridization were carried out with Dynabeads^®^ MyOne™ Streptavidin T1 (Invitrogen, #65601), and thoroughly resuspend the beads in 40 µl of water.

20 µl of the captured library were amplified, as follows: 2× KAPA HiFi HotStart ReadyMix 21μL, 5 µM primer 1μL, 20 μL captured library beads suspension. PCR amplification program was 98℃ 2 min; 98℃ 30 s; 65℃ 30 s; 72℃ 30 s, 13 cycles; 72℃ 4 min. Purifications between steps were carried out with Agencourt AMPure XP beads, and thoroughly resuspend the beads in 35 µl of water. Place the tube on a magnet to capture the beads, incubate until the liquid is clear, transfer the clear supernatant to a new tube. The libraries were estimated with Qubit dsDNA HS Assay kit (Invitrogen, Q32851).

**Sequencing**

Sample dilution, flowcell loading and sequencing were performed according to the Illumina specifications. DNA libraries were sequenced on the illumina novaseq platform as paired-end 200-bp reads.
