# Supplementary material for: Novel compound heterozygous variants in the PCCB gene causing adult-onset propionic acidemia presenting with neuropsychiatric symptoms: a case report and literature review
Source: BMC Med Genomics. 2022 Mar 16;15:59. doi: 10.1186/s12920-022-01202-2 (PMC8925091; doi:10.1186/s12920-022-01202-2)
Supplement: Supplementary file 2 — Additional file 2. The process of assessing the pathogenicity of the novel mutation for the patient. [file 12920_2022_1202_MOESM2_ESM.docx]

**Supplementary data for**

**Novel Compound Heterozygous Variants in *PCCB* Gene Causing Adult-onset Propionic Acidemia Presenting with Neuropsychiatric Symptoms: A Case Report and Literature Review**

Yingxuan Li, Miaomiao Wang, Zhaoyang Huang^*,#^, Jing Ye^*,#^ ,Yuping Wang

*: Corresponding author: Zhaoyang Huang, MD. Jing Ye, MD.

#: Authors Z. Huang and J. Ye contributed equally to this work.

This file contains the process of assessing the pathogenicity of the novel mutation for our patient. Multiple computational software programs predict that the mutation is probably a deleterious mutation. Mutation Taster, Provean, SIFT, and Polyphen-2 predicted it as disease-causing (probability 0.999), deleterious (score −4.77 < −2.5), damaging (score 0.000 < 0.05) and probably damaging (score 1.000), respectively ^[1-4](#_ENREF_1)^. And the patient’s phenotypes are highly specific for the disease propionic academia. In addition, the site of this residue is highly conserved across different species. Therefore, the novel mutation c.467T>C is classified as likely pathogenic mutation according to the standard of ACMG ^[5](#_ENREF_5)^.

**Mutation site:** PCCB c.467T>C p.I156T NM_000532.4

**Mutation taster**


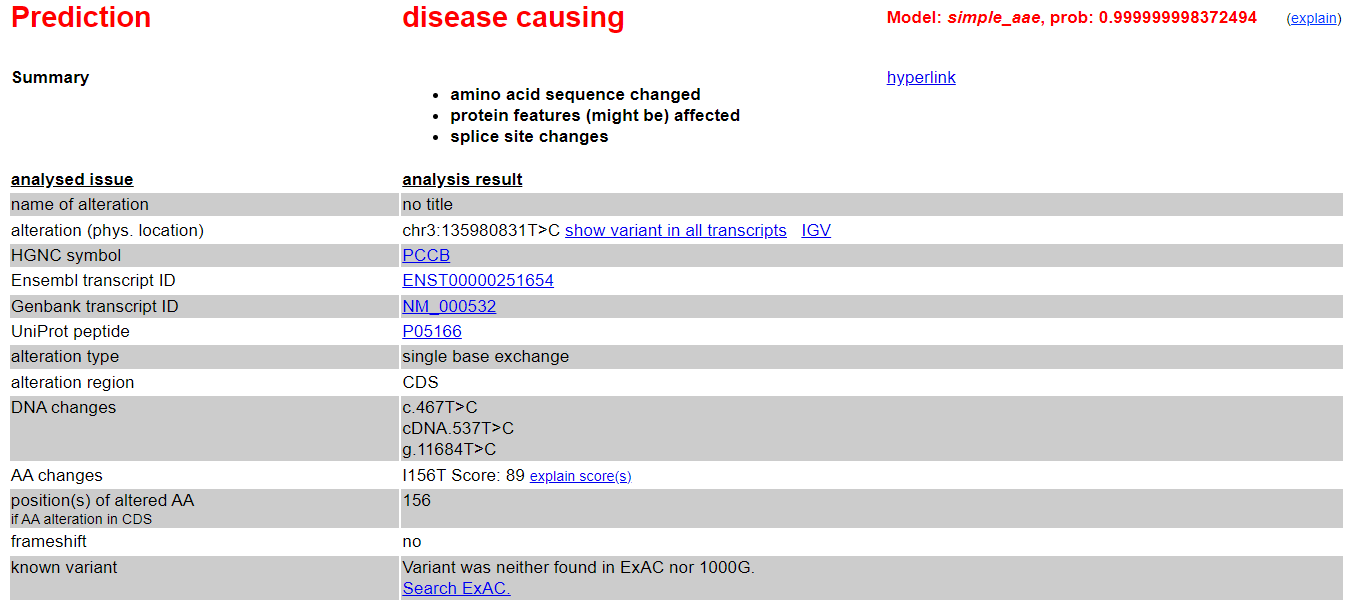


Conclusion:disease-causing (probability 0.999)

**PROVEAN & SIFT**


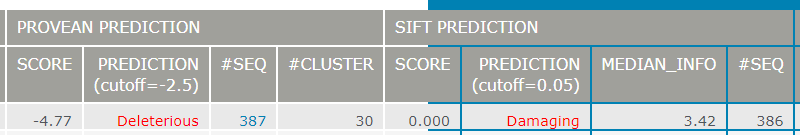


Conclusion:deleterious (score −4.77 < −2.5) and damaging (score 0.000 < 0.05)

**PolyPhen-2**


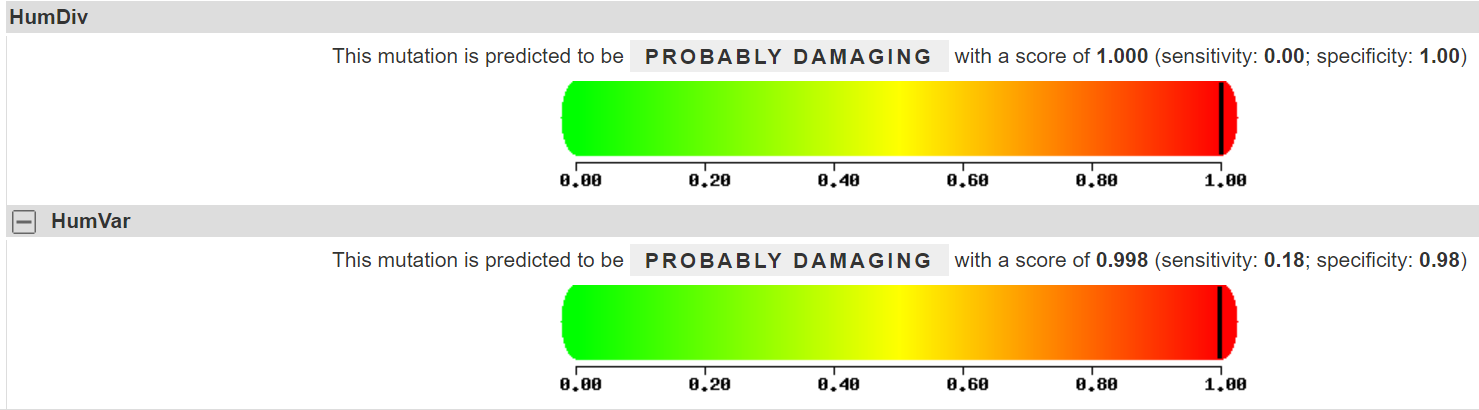


Conclusion: probably damaging (score 1.000)

**Hereditary conservation**


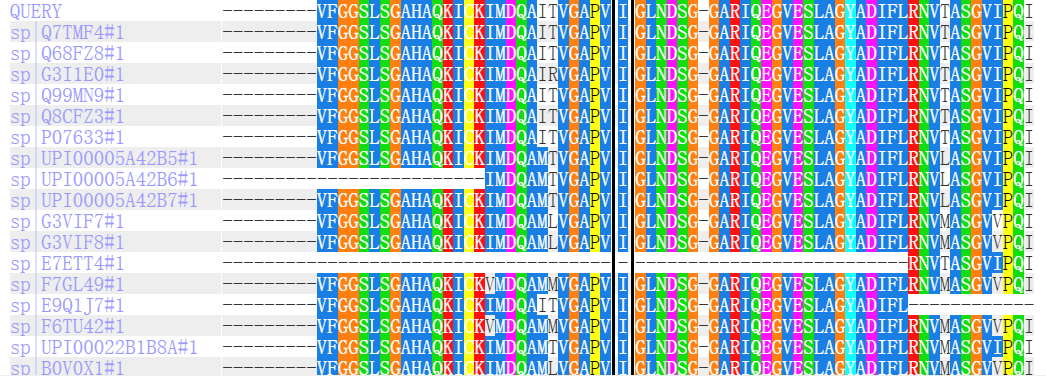


Conclusion: Highly conserved across different species. Therefore, the novel mutation c.467T>C is classified as likely pathogenic mutation according to the standard of ACMG.

**Reference:**

1. Schwarz JM, Cooper DN, Schuelke M, Seelow D. MutationTaster2: mutation prediction for the deep-sequencing age. Nature methods 2014;11:361-362.

2. Choi Y, Chan AP. PROVEAN web server: a tool to predict the functional effect of amino acid substitutions and indels. Bioinformatics (Oxford, England) 2015;31:2745-2747.

3. Kumar P, Henikoff S, Ng PC. Predicting the effects of coding non-synonymous variants on protein function using the SIFT algorithm. Nature protocols 2009;4:1073-1081.

4. Adzhubei I, Jordan DM, Sunyaev SR. Predicting functional effect of human missense mutations using PolyPhen-2. Current protocols in human genetics 2013;Chapter 7:Unit7.20.

5. Richards S, Aziz N, Bale S, et al. Standards and guidelines for the interpretation of sequence variants: a joint consensus recommendation of the American College of Medical Genetics and Genomics and the Association for Molecular Pathology. Genetics in Medicine 2015;17:405-424.
